# Reproducibility and comparison of oxygen-enhanced *T*_1_ quantification in COPD and asthma patients

**DOI:** 10.1371/journal.pone.0172479

**Published:** 2017-02-16

**Authors:** Simon M. F. Triphan, Bertram J. Jobst, Angela Anjorin, Oliver Sedlaczek, Ursula Wolf, Maxim Terekhov, Christian Hoffmann, Sebastian Ley, Christoph Düber, Jürgen Biederer, Hans-Ulrich Kauczor, Peter M. Jakob, Mark O. Wielpütz

**Affiliations:** 1 Department of Diagnostic & Interventional Radiology, University Hospital of Heidelberg, Heidelberg, Germany; 2 Translational Lung Research Center Heidelberg (TLRC), Member of the German Lung Research Center (DZL), Heidelberg, Germany; 3 Department of Experimental Physics 5, Julius-Maximilians Universität Würzburg, Würzburg, Germany; 4 Department of Radiology, Mainz University Medical School, Mainz, Germany; 5 Department of Diagnostic & Interventional Radiology with Nuclear Medicine, Thoraxklinik at University of Heidelberg, Heidelberg, Germany; 6 Comprehensive Heart Failure Center, University Hospital Würzburg, Würzburg, Germany; 7 Radiologie Darmstadt, Department of Radiology Hospital Gross-Gerau, Gross-Gerau, Germany; 8 Institute for Clinical Radiology, Ludwig Maximilians Universität München, Munich, Germany; University Children’s Hospital Bern, SWITZERLAND

## Abstract

*T*_1_ maps have been shown to yield useful diagnostic information on lung function in patients with chronic obstructive pulmonary disease (COPD) and asthma, both for native *T*_1_ and Δ*T*_1_, the relative reduction while breathing pure oxygen. As parameter quantification is particularly interesting for longitudinal studies, the purpose of this work was both to examine the reproducibility of lung *T*_1_ mapping and to compare *T*_1_ found in COPD and asthma patients using IRSnapShotFLASH embedded in a full MRI protocol. 12 asthma and 12 COPD patients (site 1) and further 15 COPD patients (site 2) were examined on two consecutive days. In each patient, *T*_1_ maps were acquired in 8 single breath-hold slices, breathing first room air, then pure oxygen. Maps were partitioned into 12 regions each to calculate average values. In asthma patients, the average *T*_1,*RA*_ = 1206ms (room air) was reduced to *T*_1,*O*2_ = 1141ms under oxygen conditions (Δ*T*_1_ = 5.3%, *p* < 5⋅10^−4^), while in COPD patients both native *T*_1,*RA*_ = 1125ms was significantly shorter (*p* < 10^−3^) and the relative reduction to *T*_1,*O*2_ = 1081ms on average Δ*T*_1_ = 4.2%(*p* < 10^−5^). On the second day, with *T*_1,*RA*_ = 1186ms in asthma and *T*_1,*RA*_ = 1097ms in COPD, observed values were slightly shorter on average in all patient groups. Δ*T*_1_ reduction was the least repeatable parameter and varied from day to day by up to 23% in individual asthma and 30% in COPD patients. While for both patient groups *T*_1_ was below the values reported for healthy subjects, the *T*_1_ and Δ*T*_1_ found in asthmatics lies between that of the COPD group and reported values for healthy subjects, suggesting a higher blood volume fraction and better ventilation. However, it could be demonstrated that lung *T*_1_ quantification is subject to notable inter-examination variability, which here can be attributed both to remaining contrast agent from the previous day and the increased dependency of lung *T*_1_ on perfusion and thus current lung state.

## Introduction

For lung diseases like chronic obstructive pulmonary disease (COPD), cystic fibrosis and asthma, global parameters available through spirometry are of limited value to monitor disease progression and treatment response on a lobar or segmental level. Non-invasive imaging methods dedicated to collect regional information on lung structure and function are considered a prerequisite for further clinical work in the field. The current clinical standard, computed tomography (CT), requires ionizing radiation and may thus be unfavorable for repeated measurements in long-term observational or interventional studies. In contrast, functional proton magnetic resonance imaging (MRI) can be repeated arbitrarily due to lack of radiation exposure [1, 2]. For instance, contrast agent-based perfusion measurements have been shown to be useful for visualizing lung function in the form of perfusion defects, exploiting the mechanism of hypoxic vasoconstriction [3, 4].

Alternatively, MRI allows for the measurement of a number of physical parameters of the investigated tissue, among which the *T*_1_ relaxation time appears particularly interesting in the lungs [5–8]: *T*_1_ depends on a number of morphological and functional parameters, including tissue composition and blood volume content. Importantly, since molecular oxygen (*O*_2_) is paramagnetic, it reduces *T*_1_, connecting this reduction to local ventilation. While most published work on COPD and asthma patients is based on the visual detection of *T*_1_-weighted signal intensity changes induced by inhalation of pure oxygen [9, 10], others employed quantification of *T*_1_ itself to produce potentially useful diagnostic information on regional lung ventilation and state [11–14]: *T*_1_ and the oxygen-induced *T*_1_ reduction were found to be significantly different in diseased areas of the lung and correlate with the GOLD stage in COPD patients. Since these approaches utilize only pure oxygen as an endogenic contrast agent or, when considering room air *T*_1_ maps alone, no agent at all, they appear very well suited for imaging that accompanies therapy in a clinical setting. The fast quantification methods developed for *T*_1_ mapping in the lungs can also be completed in very short breath-holds (≈ 6s) suitable for dyspnoeic patients.

For all forms of functional MR imaging, a primary goal of parameter quantification is to gain absolute values reflecting the physical characteristics of the tissue independent of the scanner environment being used. Such parameter mapping can be repeated at regular intervals and the measured values can be compared both between subjects and the same subject at different timepoints, which would be an advantage for longitudinal monitoring. Given the epidemiologic and economic importance of asthma and COPD, a suitable tool for functional lung imaging in these patients would be highly appreciated as biomarker for current and future research. However, MRI-based parametrization of lung tissue in COPD patients is particularly challenging due to the inherently lower lung signal in emphysematous lungs. Accordingly, the aims of this study were to confirm previously reported data on the characteristic lung *T*_1_ values found in patients with COPD, provide comparable data for asthmatics in contrast to healthy subjects, and to investigate the intra-individual reproducibility of these values in repeated measurements.

## Materials and methods

### Patient selection

The study was carried out as part of a prospective trial (German Clinical Trials Register number DRKS00005072) approved by the institutional ethics committee, and conducted according to the recommendations of the review board. The study was approved by the Institutional Review Board of the Medical Faculty of the University of Heidelberg, Germany. All subjects gave written informed consent for examination and data evaluation. The work was carried out in accordance with The Code of Ethics of the World Medical Association (Declaration of Helsinki). Patients were included based on clinical diagnoses as indicated by spirometry and [15] effectively divided into three groups: 12 patients with asthma and 12 patients with COPD were examined at site 1, further 15 COPD patients at site 2. The COPD patient group included GOLD [16] stages I to IV. Exclusion criteria were recent exacerbations, inability to hold breath for 10s and any contraindication for MR imaging.

### MRI measurements

All measurements were performed on clinical 1.5T scanners (Magnetom Avanto, Siemens Medical Solutions, Erlangen, Germany) equipped with identical hard- and software at both study sites. An Inversion Recovery (IR) SnapShot FLASH sequence [6] was used in 8 coronal slices in the lungs of each patient, during one 6s expiratory breath-hold for each slice. Each measurement consists of 32 individual images, acquired with a short echo time of TE = 750 *μ*s using an asymmetric readout to compensate for the short $T_{2}^{*}$ in lung tissue. At *TR* = 3ms, the time resolution for each image was 192ms with a matrix size of 64 × 128 in a 50 × 50cm^2^ field of view and 15mm slices. The flip angle was chosen as *α* = 8°, corresponding with the effective Ernst angle in the lungs for an expected *T*_1_ ≈ 1100ms.

This *T*_1_ quantification experiment was repeated four times: First, under normoxic conditions, i.e. at 21% oxygen (*O*_2_) in the breathing gas. Then, using a standard clinical oxygen mask, 100% *O*_2_ was supplied at 15l/min to introduce hyperoxic conditions. After 3min to allow for a complete wash-in of oxygen [8], the entire experiment was repeated. The entire procedure was repeated on the following day, on average (22.9 ± 0.9)h later. This *T*_1_ quantification experiment was embedded in a full protocol of morphological MRI sequences and followed by a functional DCE perfusion measurement that included the injection of an MRI contrast agent as reported previously (0.1 mmol/kg body weight Gd-DTPA, Magnevist, Bayer Schering Pharma AG, Berlin, Germany) [14, 17]. The total duration of the protocol was about 30min for each patient.

Prior to the patient measurements, *T*_1_ mapping was performed using a specifically designed phantom and the same healthy volunteer on both used scanners [18] to ensure no discrepancies were introduced by the MRI equipment.

### Data analysis and statistics

**Fig 1:**
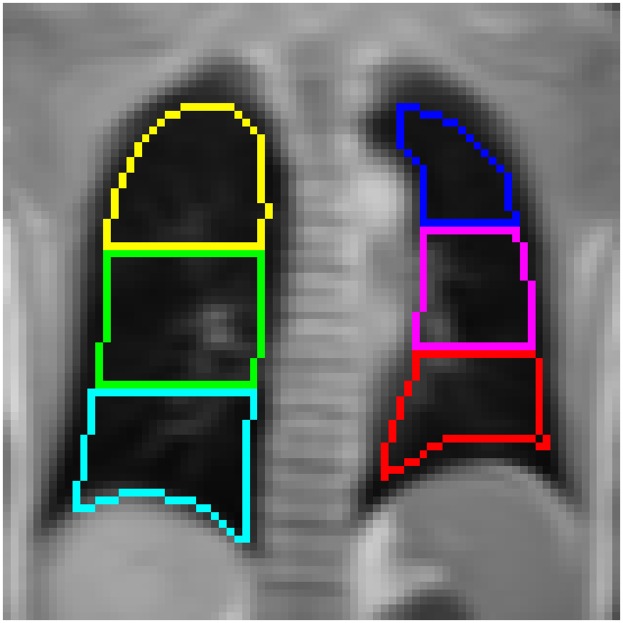
Anterior regions of interest in an asthma patient, generated from a manually drawn mask. ROIs are shown in different colours.

*T*_1_ maps were calculated using an exponential parameter fit implemented to run directly on the scanner, producing parameter maps alongside the MR images according to [19]. To find *T*_1_ values for the lungs, masks were drawn manually by a single observer to select the lungs on the acquired slices. To account for the large inhomogeneity of *T*_1_ in diseased lungs (as shown below), these masks were then separated by software into 12 regions for each patient, providing upper, middle and lower regions for both lungs, each divided into an anterior and posterior volume. This masking is illustrated for one patient in Fig 1. Software to assist with masking and to perform the regional separation was written in MATLAB (Matworks, Natick, MA). Both median *T*_1_ values for entire lung volumes and the median *T*_1_ in these regions were calculated. As a measure of *T*_1_ inhomogeneity, an intra-patient standard deviation *σ*_*T*1_ for both room air and oxygen measurements was determined, from the median values within all regions.

$\Delta T_{1}=\frac{T_{1,RA}-T_{1,O2}}{T_{1,RA}}$, the relative reduction of *T*_1_ due to *O*_2_ was also determined from *T*_1_ at normoxic and hyperoxic conditions. For the reduction of *T*_1_ due to oxygen in each group as well as the combined group of COPD patients from both sites, *p*-values were calculated according to a Wilcoxon signed-rank, as a normal distribution of values could not be assumed in this small sample size. For the difference in baseline *T*_1_ in the asthma and COPD groups, a Wilcoxon rank-sum test was used to calculate *p*-values. Finally, *T*_1_ measured on day 1 and day 2 was compared using the method of Bland and Altman [20]. Values of *p* < 0.05 taken from these tests were considered statistically significant.

## Results

**Fig 2:**
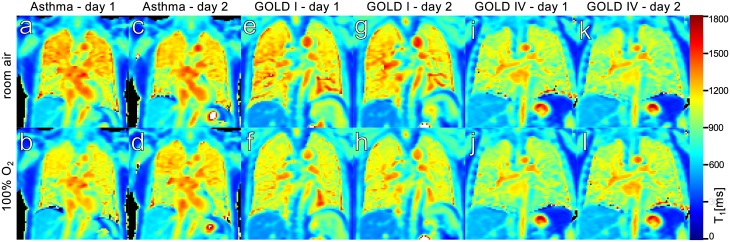
Example *T*_1_ maps, acquired in one asthma (a-d) and two COPD (g-k) patients from different GOLD stages. The maps in the upper row were measured at 21%O_2_ in the breathing gas, the lower row during administration of 100%O_2_. For each patient, *T*_1_ maps from equivalent slices on both measurement days are shown to illustrate reproducibility.

Fig 2 shows *T*_1_ maps acquired in three different patients. The instances of fairly homogenous *T*_1_ distribution in the lungs, as in Fig 2i–2l ut also highly inhomogenous cases such as in Fig 2a–2d, were the motivation for the masks drawn over the entire lungs to be split into regions as described above. To illustrate the comparability of slice and breathing position for the reproducibility measurements, parameter maps on concurrent days are shown as well. While notable inhomogeneity was also present in many of the COPD patient *T*_1_ maps, on average significantly shorter *T*_1_ values were prevalent compared to the asthmatics.

Table 1 shows median *T*_1_ values for in the entire lungs sorted by pathologies and sites. Considering the switch from room air to pure oxygen, statistically significant reductions were observed in all groups. Note that the standard deviations given in Table 1 are for the inter-patient variance of *T*_1_. Due to the large inhomogeneity of *T*_1_ in the examined pathologies, the average intra-patient standard deviation was *σ*_*T*1,*RA*_ = 141ms among the regions in each COPD patient’s lung and *σ*_*T*1,*RA*_ = 102ms in asthma patients. Comparing these average *T*_1_ for all patients in a Wilcoxon rank-sum test gives *p* < 10^−3^ for the statistical difference between asthma and all COPD patients (with *p* < 10^−2^ for the COPD measurements from single sites alone) but at *p* = 0.79 no statistical significance for the difference between the COPD measurements at different sites.

**Table 1:** Median *T*_1_ values over the entire lungs of patients.

		Day 1				Day 2			
	*n*	*T*_1,*RA*_ [ms]	*T*_1,*O*2_ [ms]	Δ*T*_1_	*p*	*T*_1,*RA*_ [ms]	*T*_1,*O*2_ [ms]	Δ*T*_1_	*p*
Asthma site 1	12	1206 ± 63	1141 ± 65	5.4%	4.9 ⋅ 10^−4^	1186 ± 43	1123 ± 35	5.2%	4.9 ⋅ 10^−4^
COPD site 1	12	1125 ± 64	1086 ± 62	3.4%	9.8 ⋅ 10^−4^	1110 ± 58	1056 ± 48	4.9%	4.9 ⋅ 10^−4^
COPD site 2	15	1124 ± 71	1077 ± 66	4.2%	6.1 ⋅ 10^−5^	1086 ± 81	1040 ± 74	4.2%	6.1 ⋅ 10^−5^
COPD sites 1&2	27	1125 ± 67	1081 ± 63	3.9%	6.3 ⋅ 10^−6^	1097 ± 71	1047 ± 63	4.5%	5.6 ⋅ 10^−6^

Asthma patients and COPD patients at both sites are listed separately as well as all COPD patients together. Values are given for each examination day separately, including the relative reduction due to the breathing of pure oxygen and *p*-values for the significance of this reduction found through a paired *t*-test.

**Fig 3:**
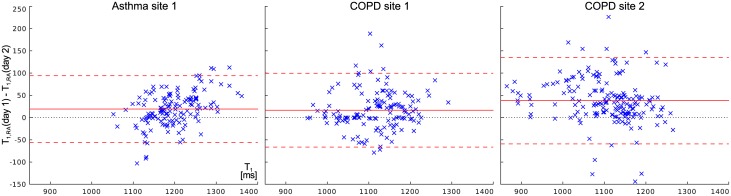
Bland-Altman plots comparing the difference in *T*_1_ under room air conditions measured at day 1 and day 2 to the average of both days. Data from asthma and COPD patients examined at both sites is shown. The average difference is shown as a solid line and 95% confidence intervals (1.96*σ*) as dashed lines.

Fig 3 contains Bland-Altman plots [21] relating the difference between the *T*_1_ measurements on consecutive days to the mean of both measurements, calculated from the median *T*_1_ values taken from lung regions. As seen in Table 1, *T*_1_ measured at the second day was found to be shorter on average in all patient groups. Notably, this systematic discrepancy is more prononounced at site 2. However, with a 95% confidence interval of 90ms, the inter-patient variance of this difference in the repeat measurements is large compared to the average of −26ms. This is equal to average relative differences of 2.2% for asthma and 2.1% for COPD patients at site 1 and 3.6% among COPD patients at site 2.

**Fig 4:**
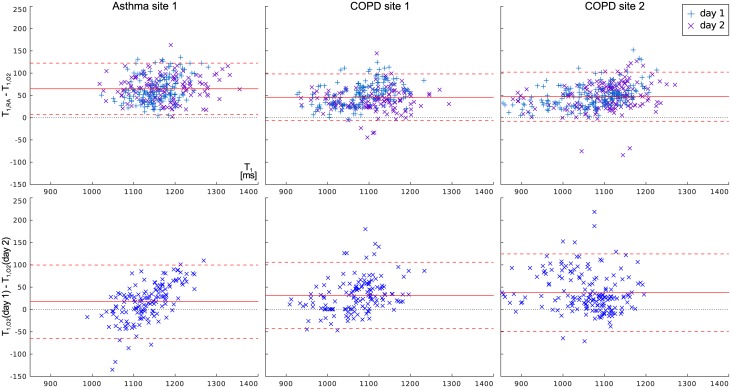
Bland-Altman plots relating the *T*_1_-reduction due to pure oxygen to the mean of both *T*_1_ values. The lower row shows the reproducibility of *T*_1_ under hyperoxic conditions on both days, analogous to Fig 3.

The observed effect on *T*_1_ of switching the breathing gas from room air (21% *O*_2_) to 100% *O*_2_ is displayed in Fig 4. The absolute difference in *T*_1_ due to *O*_2_ is shown with per-region median values for both days. While the reduction is larger in the asthma patients, it appears very similar in both groups of COPD patients.

Finally, the repeat measurements of *T*_1_ under hyperoxic conditions also shown in Fig 4 again display a systematic tendency to shorter *T*_1_ on the second day. With a 95% confidence interval of 85ms and average difference of −30ms, the errors in the hyperoxic *T*_1_ measurements are similar to those of the base *T*_1_, even though they include an additional imprecision introduced by the difficulty of achieving the same oxygen concentration in the breathing gas on both days. The absolute relative variation of the oxygen-induced Δ*T*_1_ from day to day was found to be 23% for asthma and 30% for COPD. As all measurement errors contribute to this value, it is the least precise parameter produced in this study.

## Discussion

The average *T*_1_ values measured in the lungs of asthma and COPD patients in this study were found to be significantly shorter than values previously reported in healthy subjects, which tend to be between 1170ms and 1300ms [6, 8, 22]. Notably, average *T*_1_ in asthma lies between the values for COPD and healthy subjects. This can be attributed to the effective lung *T*_1_ being comprised of the longer *T*_1_ provided by the blood fraction and a shorter *T*_1_ component by the alveolar structure [23]. Accordingly, the reduced *T*_1_ in diseased tissue likely reflects both reduced perfusion and pathological changes in the pulmonary tissue. This is also apparent in that the average standard deviation of *T*_1_ within the lungs as a measure of inhomogeneity is higher in COPD than in asthma, even though *T*_1_ itself is shorter. This further confirms the necessity for regional assessments of lung disease with quantitative imaging methods.

The precision of *T*_1_ quantification in diseased lungs appears to suffer from reduced SNR due to low proton density. The large inhomogeneity *T*_1_ within the lung volume caused by the varying degrees of emphysemal destruction itself also increases the need to ensure identical ROI and breathing state positions. However, even though it is considerably weaker than in healthy volunteers, the *T*_1_ reduction due to pure oxygen is almost always visible and statistically highly significant over the study participants. Notably, *T*_1_ quantification under hyperoxic conditions additionally loses accuracy since the administration of *O*_2_ gas with conventional clinical masks is somewhat unreliable: The actual oxygen concentration will likely be less than 100% and vary slightly due to the adjustment of the breathing mask [24]. Nevertheless, statistically significant reductions of *T*_1_ due to oxygen administration were found in all patient groups in this study and with 54ms and 56ms for COPD at sites 1 and 2 and 59ms for asthma patients, the 95% confidence intervals for the distribution of the reduction shown in Fig 4 are very similar. Interestingly, this reduction was on average lower than the 8% to 12% previously reported in healthy subjects [6, 8], confirming the diagnostic relevance of oxygen enhancement in chronic obstructive lung diseases [14].

As the lung masks were drawn manually, they may be inherently biased by observer input, limiting the parameter quantification. A fully automatic segmentation of images would be desirable to ensure unbiased reproducible measurements. Apart from the imprecision of *T*_1_ quantification itself, a notable, though at the small patient numbers investigated here not statistically significant, lowering of *T*_1_ from the first experiment day to the second was observed in this study. Several sources for this discrepancy can be proposed: Patients may become accustomed to the breathing commands given during the study protocol, leading to deeper exhalation on the second day. However, while on average smaller lung volumes were found on the second day, no correlation between volume and *T*_1_ reduction was found (this is illustrated in S1 Fig). While in earlier publications, an effect of respiratory state on lung *T*_1_ had been found [12], in more recent work no such dependency was found [25, 26]. In contrast, when observing oxygen-induced signal enhancement as a measure of ventilation, the change in proton density due to respiratory state may be an issue.

A very probable source for *T*_1_ reduction is remaining contrast agent that was injected during the later steps in the study protocol during the first day: The mean serum elimination half-life of the employed 0.1mmol/kg Gd-DTPA dose is 1.6±0.1h [27] for healthy subjects with normal renal function. At this rate, less than 0.005% of the original dose would remain after 23h and no effect on lung *T*_1_ should be measurable. However, chronic renal impairment commonly occurs in patients with COPD [28] and even though the study subjects were screened to have glomerular filtration rates (GFR) of at least 40ml/min, the elimination half-life of Gd-DTPA has been shown to increase to 4.2±2.0h at clearance rates between 30ml/min and 60ml/min [27]. Thus, assuming a relaxivity of 4.1l/s/mmol of Gd-DTPA in blood [29], the *T*_1_ shortening from 1124ms to 1086ms observed in COPD patients at site 2 could be fully explained by remaining contrast agent if the mean elimination half-life is as long as 3.65h. Notably, this both requires significant renal impairment to be prevalent within the patient collective and provides no explanation for the larger reduction on site 2 in comparison to site 1, as contrast agent application was identical on both sites to ensure comparability of perfusion measurements and thus likely accounts for only part of the discrepancy in *T*_1_. A change in the experiment setup can be discounted entirely, since measurements were distributed over a long period with repeat on consecutive days. As such, changes in circumstances would at best affect different patients but not intra-patient repeat measurements.

Finally, as stated above, *T*_1_ in the lungs, especially in patients, is strongly affected by perfusion which is in turn influenced by ventilation through hypoxic vasoconstriction [2–4, 30]. This means that even short-term influences on lung function may have an effect on the observable *T*_1_, which is after all what the measurement is intended for. Though changes of therapeutic treatments between the two MRI sessions were avoided, the study protocol includes both a large number of very short breath-holds and an administration of pure oxygen, which comes up to physical therapy for the patients. As such, the observed site-dependent lowering of *T*_1_ on consecutive days both highlights the difficulty to achieve repeatable measurements and the sensitivity of the *T*_1_ quantification to changes in lung vital status.

In its entirety, this study emphasizes multiple difficulties in *T*_1_ mapping in COPD and asthma patients: The IRSnapShot FLASH sequence as employed here requires only very short breath-holds, but suffers from the low proton density in emphysematous tissue and unsteady depth of repeated breath-holds. The short measurement times determined by *T*_1_ relaxation also limit the amount of signal that can be acquired. To adress these challenges, MRI sequences that improve on the basic IRSnapShot FLASH by employing ultrashort echo times during free breathing [31, 32] or a balanced steady-state fast precession (bSSFP)-based readout [26] have been demonstrated and applied to COPD [25], though the reproducibility of these methods in patients remain to be tested.

## Conclusion

In this work, the characteristically short lung *T*_1_ values previously reported in COPD were confirmed along with smaller Δ*T*_1_ induced by the administration of pure *O*_2_ than commonly observed in healthy subjects. In addition, an average *T*_1_ in asthma patients was found to lie between values typical for healthy volunteers and COPD patients. The lung *T*_1_ values found display both large inter-patient and intra-patient variations, with inhomogenous *T*_1_ within the lungs also being distinctive for diseased lungs. In the reproducibility measurements, relevant variability within data from day 1 was found, for the first time presenting a range of measurement variation for *T*_1_ values in the diseased lung in the short term, but also underlining the sensitivity of *T*_1_ mapping to physiological conditions. The possible influence of remaining contrast agent on the repeated *T*_1_ measurements also highlights the need to consider time intervals for quantitative measurements within longitudinal studies with the specific pathology in mind: An interval that is reasonable for healthy volunteers may be insufficient for patients with impaired renal function.

## Supporting information

S1 Fig Correlation of ROI areas and *T*_1_ differences. a: Bland-Altman plot of the number of voxels *n*_*v*_ in each ROI at both measurement days. b: The relative change in *n*_*v*_ compared to the relative change in *T*_1_ from day 1 to day 2.
(EPS)
